# EMP3 mediates glioblastoma‐associated macrophage infiltration to drive T cell exclusion

**DOI:** 10.1186/s13046-021-01954-2

**Published:** 2021-05-08

**Authors:** Qun Chen, Jing Jin, Xin Huang, Fan Wu, Hongguang Huang, Renya Zhan

**Affiliations:** Department of Neurosurgery, The First Affiliated Hospital, Zhejiang University School of Medicine, #79 Qingchun Road, 310003 Hangzhou, Zhejiang Province P.R. China

**Keywords:** Glioblastoma, Epithelial membrane protein 3, Exhausted T cells, Tumour‐associated macrophages

## Abstract

**Background:**

The immunosuppressive tumour microenvironment is a critical factor in the initiation and progression of glioblastoma (GBM), which is characterized by an abundance of tumour-associated macrophages (TAMs) but a paucity of infiltrating T cells. In this research, we studied whether epithelial membrane protein 3 (EMP3) plays a crucial role in immune modulation in GBM.

**Methods:**

TCGA and CGGA transcriptomic profiles of wild-type IDH1 GBM were used for bioinformatic analysis. The role of EMP3 in GBM was validated through *in vivo* and *in vitro* experiments. Human GBM specimens were collected and evaluated using immunofluorescence analysis.

**Results:**

EMP3 was associated with immunosuppression in GBM. Elevated EMP3 in GBM areas was accompanied by high expression of PD-L1 and abundant M2 TAM recruitment but a lake of T cell infiltration. We found that EMP3 was a potent protein in M2 TAM polarization and recruitment that impaired the ability of GBM cells to secrete CCL2 and TGF-β1. Furthermore, EMP3 suppressed T cell infiltration into GBM tumours by inhibiting the secretion of CXCL9 and CXCL10 by macrophages and led to an effective response to anti-PD1 therapy.

**Conclusions:**

EMP3 is thus a critical immunosuppressive factor for recruiting TAMs in GBM and suppressing intratumoural T cell infiltration to facilitate tumour progression and is a potential therapeutic target.

**Supplementary Information:**

The online version contains supplementary material available at 10.1186/s13046-021-01954-2.

## Background

GBM is one of the most malignant brain tumours. Although patients receive active treatment, the 5-year survival rate is less than 5 %. GBM is characterized by a lack of T cell infiltration but abundant tumour-associated macrophage (TAM) infiltration [1]. M2 TAMs are the main TAMs in most malignant tumours and play important roles in immunosuppression and treatment resistance [2, 3]. In addition, there is a strong association between poor survival and increased M2 TAM infiltration within the tumour microenvironment (TME) of human GBM patients. M2 TAMs are recruited to the TME and are emerging as important mediators of adaptive resistance to immune attack. However, how M2 TAMs polarize and inhibit antitumour immunity within GBM is still unknown. Loss of effector functions and increases in immunosuppressive molecules are the major characteristic features of exhausted T cells. In fact, in GBM patients, it is common for the numbers of CD4^+^ and CD8^+^ T cells to decrease within the tumour and in the circulation [4, 5]. With the expansion of indications for checkpoint inhibitors, an increasing number of patients are receiving these drugs. Despite the durable clinical response to antibodies that block PD-1 seen in advanced melanoma, non-small cell lung cancer (NSCLC), renal cell carcinoma, and other cancers, it is not clear whether this treatment is feasible in GBM [6]. Indeed, the exhaustion mechanism of T cells in GBM should be further studied.

It has been reported that epithelial membrane protein 3 (EMP3) is a tetraspanin membrane protein that represses the induction and function of cytotoxic T lymphocytes [7]. The expression of EMP3 is low in the adult brain. However, EMP3 expression is upregulated in brain tumours, especially in GBM. The expression of EMP3 mRNA has been shown to be associated a with poor outcome [8–11]. Depletion of the EMP3 protein attenuates malignant behaviours of GBM cells [12]. Our preclinical studies demonstrated that EMP3 drives the infiltration of M2 TAMs into GBM, suppresses CD4^+^ and CD8^+^ T cell infiltration, and has the potential be exploited as a therapeutic target.

## Methods and materials

### Data collection

Publicly available clinical data for GBM patients with wild-type IDH1 (IDH1wt) and their transcriptomic data were obtained from the TCGA (https://cancergenome.nih.gov/) and CGGA (http://www.cgga.org.cn/) databases. GBM patients with IDH1wt sequence-based gene expression (*n* = 152) and array-based gene expression (*n* = 475) data form the TCGA dataset were used. GBM patients with IDH1wt array-based gene expression data (*n* = 94) from the CGGA dataset were used.

### Bioinformatic analysis

Downloaded GSEA software version 3.0 (www.broadinstitute.org/gsea/) was used for GSEA, which was used to calculate whether a gene set exhibited statistically significant [13] concordant differences between two defined groups (high expression of EMP3 versus low expression of EMP3) in the TCGA array-based gene expression dataset. ssGSEA, an extension of GSEA, was used to calculate separate enrichment scores for each pairing of a sample and gene set online (https://www.genepattern.org/) [14].

### Cell transfection

EMP3 lentiviruses were purchased from GeneChem (Shanghai, China). A CRISPR/Cas9 system was employed to knock out EMP3 in GL261 cells. According to our previous research [15], cells were transfected with LV-Cas9 lentiviruses at an MOI of 8 for 72 h and selected with puromycin at a final concentration of 3 µg/mL for 7 days. GL261 cells were subsequently infected with lentiviruses containing sgRNA targeting EMP3. The expression of EMP3 was confirmed by Western blot after infection with the sgRNA for 4 h. The lentiviruses expressing Cas9, sgRNAs and negative control (LV-NC) were constructed by GeneChem (Shanghai, China). The sequences of the sgRNAs are shown in Supplementary Table 1.

### Cell lines

GL261, RAW 264.7, and BV-2 cells were purchased from the Chinese Academy of Sciences Cell Bank in 2018 and maintained in Dulbecco’s modified Eagle’s medium (DMEM) (Sigma-Aldrich) supplemented with 10 % foetal bovine serum (FBS, HyClone). The Lonza Mycoalert Mycoplasma Detection Kit (Lonza, Switzerland) was used to test for mycoplasma contamination. All cells were cultured at 37 °C in an atmosphere of 5 % CO_2_ and maintained in culture for less than 20 passages.

### Western blot analysis

Protein was extracted from GL261 cells using lysis buffer containing RIPA buffer (Solarbio, 89,901) and a protease inhibitor (Solarbio, A8260). The BCA Protein Assay Kit (Solarbio, PC0020) was used to test the protein concentration according to the manufacturer’s instructions, and 20 µg/lane protein was loaded per well. The primary antibodies used were anti-EMP3 (1:1000, Abcam, 236,671) and anti-β-actin (1:1000, CST, 4970). PVDF membranes were incubated with primary antibodies overnight at 4 °C and then incubated with HRP-conjugated anti-rabbit IgG (1:10,000, Sigma-Aldrich, RABHRP1) or HRP-conjugated anti-mouse IgG (1:10,000, Sigma-Aldrich, RABHRP2) at 37 °C for 1 h. The protein bands were visualized using an Immobilon Western Chemilum HRP Substrate kit (Millipore, WBKLS0050) and Bio-Rad Gel Doc XR imaging system (Bio-Rad, USA).

### Chemotaxis assay

Briefly, T cells (5 × 10^5^) were isolated from the spleen and lymph nodes of C57BL/6 mice. Anti-CD3 beads (Miltenyi Biotec) were used to purify the T cells. Subsequently, the T cells were labelled with CFSE and seeded in 24-well plates (5-µm pore size, Corning). Supernatants from RAW264.7 cells treated with 50 U/mL IFN-γ, 10 µg/mL anti-CXCL9 neutralizing antibodies, and/or 10 µg/mL anti-CXCL10 neutralizing antibodies (R&D Systems) were placed in the bottom chambers. The whole system was incubated for 48 h at 37 °C in an atmosphere of 5 % CO_2_. A flow cytometry assay was performed to count the cells in the bottom chamber. Chemotactic index = (migrated cells – spontaneously migrated cells) /total T cells plated in the Transwell × 100 % [16].

### Migration assay

Migration assay was performed to assess RAW 264.7 cell migration in 24-well plates (8-µm pore size, Corning). Briefly, RAW 264.7 cells (5 × 10^4^) were planted in the upper chambers which has been precoated with Matrigel (Corning). The lower chamber was placed with supernatants from GL261 cells with different treatment. The whole system was incubated for 48 h at 37 °C in an atmosphere of 5 % CO_2_ for RAW 264.7 cell migration. The cells were stained with trypan blue. All images were processed and analyzed using ImageJ.

### Immunofluorescence

Neoplastic tissues were collected from GBM patients and syngeneic intracranial GBM models, fixed with formalin, and embedded in paraffin. Frozen sections tumours were from obtained syngeneic intracranial GBM models. Paraffin-embedded sections and frozen sections were used for immunofluorescence staining. Primary antibodies, including anti-PD-L1 (Abcam, #224,030), anti-EMP3 (Invitrogen, PA5-97705), anti-CD86 (Abcam #239,075), anti-CD206 (Abcam, #125,028), anti-CD8α (Invitrogen, A700-044), anti-CD4 (Invitrogen, MA5-32166), and anti-Ki67 (CST, #12,075), were diluted (1:100) in PBS with 1 % BSA. After overnight incubation at 4 °C, the samples were washed three times with PBS and incubated with FITC-labelled anti-IgG (Alexa Fluor 488 or 594, Thermo Fisher) for 2 h at room temperature. DNA was stained with DAPI (Sigma-Aldrich, USA), and samples were visualized with a fluorescence microscope (Olympus, Japan). The proportion of stained cells/field was subjected to statistical analysis. All images were processed and analysed using ImageJ.

### Haematoxylin and eosin (H&E) staining

Paraffin-embedded sections were used for H&E staining. Briefly, deparaffinized and rehydrated brain sections on slides were incubated with haematoxylin (Sigma-Aldrich) for 4 min to stain the nuclei and then incubated with eosin (Solarbio) for 2 min.

### Measurement of cytokine and chemokine levels

RAW264.7 cells were cultured in 96-well plates for 24 h, culture supernatants were collected, and the secretion of TNF-α and IL-10 was detected with an Inflammatory Cytometric Bead Array kit (BD Biosciences) according to the manufacturer’s instructions. GL261 cells were cultured in 96-well plates for 24 h, culture supernatants were collected, and the secretion of TGF-β1 and CCL2 was measured with ELISA kits purchased from RayBiotech following the manufacturer’s instructions.

### RNA extraction and real‐time quantitative PCR (qRT-PCR)

Total RNA was isolated from RAW264.7 and GL261 cells using TRIzol reagent according to the manufacturer’s instructions (Invitrogen). Total RNA and the PrimeScript RT Reagent Kit (Takara, Japan) were used to synthesize cDNA. qRT-PCR was performed with a reaction mixture containing SYBR Green (Takara, Japan) for triplicate samples with the CFX96 Touch Real-Time PCR Detection System (Bio-Rad, USA) [17]. The expression levels of genes were normalized to that of β-actin through the 2^−ΔΔCt^ method. The primer sequences used for PCR in this study are listed in Supplementary Table 2.

### Patients

The GBM tissues used in this study were from 27 patients. Patient information is listed in Supplementary Table 3. All the patients included in the study were from The First Affiliated Hospital, Zhejiang University School of Medicine. All research performed was approved by the Clinical Research Ethics Committee of the First Affiliated Hospital, Zhejiang University School of Medicine. The enrolment criteria were as follows: (1) 18- to 70-year-old male or female; (2) primary GBM diagnosed by a pathologist; (3) wild-type status for IDH1 in GBM tissues; and (4) voluntary participation in the study with a signed informed consent form (ICF). The exclusion criteria were as follows: (1) unwilling to participate in the study and refused to sign an ICF; and (2) other conditions considered by the researchers to be inappropriate for inclusion. The clinical data are listed in Supplementary Table 3.

### Syngeneic intracranial GBM model

Four-week-old female C57BL/6 mice were purchased from Beijing Vital River Laboratory Animal Technology Co., Ltd. (China). Briefly, a total of 4 × 10^5^ GBM cells (GL261 EMP3_KO and GL261 EMP3_Scra) were injected into the brain of each mouse using stereotactic equipment. The intracranial tumours were measured with bioluminescence imaging on days 7, 21 and 35, and the survival time was recorded until the experimental endpoints. Mice were injected intraperitoneally with an anti-CXCR3 mAb (200 µg per mouse, BD Biosciences) or IgG (200 µg per mouse, BioXCell) on the first day after tumour implantation and then every 3 days for a total of 6 doses. An anti-PD-1 antibody was purchased from Merck. After tumour implantation, mice were treated with PD-1 blockade (200 µg per mouse, Selleck) or IgG (200 µg per mouse, BioXCell) every 4 days for a total of 5 doses. All animal experiments were approved and supervised by the Tab of the Animal Experimental Ethical Inspection of the First Affiliated Hospital, Zhejiang University School of Medicine.

### Ex vivo analysis of the infiltration of T cells by multicolour FACS

Mice were euthanized for ex vivo immune analysis. The spleen, brain, and blood were collected for flow cytometry. Lymphocytes isolated from brain tumours were purified using Percoll or Ficoll gradient centrifugation. Single-cell splenocyte suspensions were obtained using mechanical dissociation of the spleen followed by RBC lysis. CD4^+^ and CD8^+^ T cells were gated from CD3^+^ T cells. For subtype staining, CD4^+^ and CD8^+^ T cells were fixed and permeabilized with a Fix & Perm kit (BD Biosciences) and stained for intracellular cytokines. The following antibodies were used in the analysis: anti-IFN-γ (XMG1.2), anti-TNF-α (MP6-XT22), and anti-IL-2 (JES6-5H4), which were purchased from BD Biosciences. Flow cytometric analysis was conducted on a FACSFortessa flow cytometer (BD Biosciences), and FlowJo software (FlowJo, LLC) was used to analyse the data.

### Statistics

The TCGA and CGGA transcriptomic data were processed through CIBERSORT (https://cibersort.stanford.edu/) using the default LM22 immune cell gene signatures. Pearson’s correlation was applied to correct for two comparisons. Confidence intervals for the Pearson’s correlation coefficients were calculated using the normal approximation given by the Fisher Z-transformation. The Kaplan-Meier method was used to generate survival curves. The log-rank test was used to assess statistical significance between different groups. Differences between groups were analysed using an unpaired two-tailed t-test. ROC (receiver operating characteristic) curves were generated by using SPSS 22.0. R version 3.3.2, with the extension package “corrplot” used to produce figures. Data are presented as the mean ± SD, and all *in vitro* experiments were replicated at least 3 times. p < 0.05 was considered statistically significant.

## Results

### EMP3 is associated with immunosuppression in GBM

**Fig. 1:**
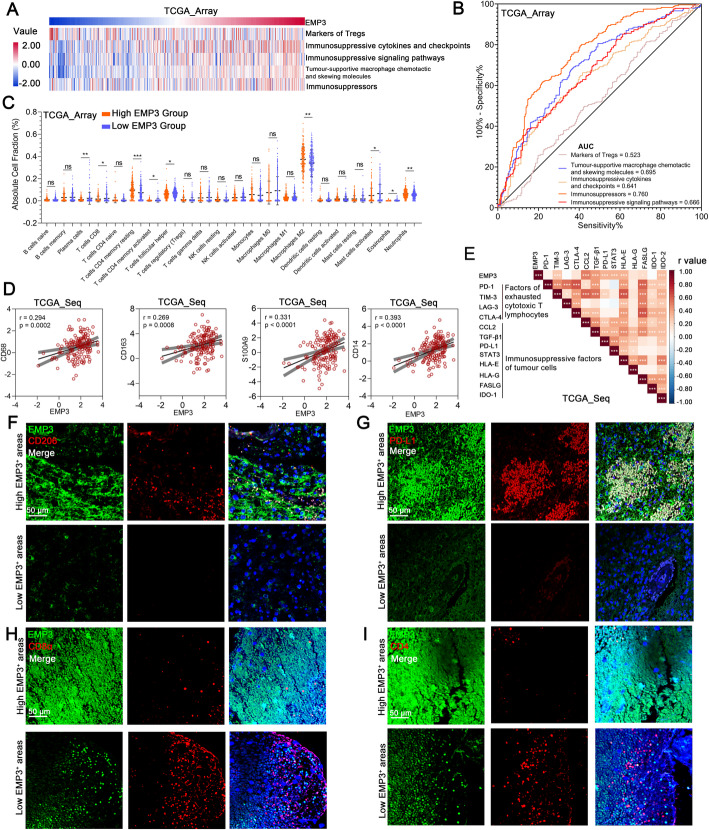
EMP3 is associated with immunosuppression in GBM. **a** Pearson correlation analysis of the expression of EMP3 with GBM-associated immunosuppressive metagenes in the TCGA_array GBM dataset (*n* = 475). **b** ROC curves indicated that high EMP3 was involved in immunosuppression in the TCGA_array GBM dataset (*n* = 475). **c** Immune cell fractions were estimated using CIBERSORT, and the differences between the cell fractions of the high and low EMP3 groups in the TCGA_array GBM dataset were evaluated using Student’s t-test (*n* = 475). **d** Pearson correlation plots of the expression of EMP3 with M2 TAM signature markers (CD68, CD163, S100A9, and CD14) in the TCGA_array GBM dataset (*n* = 475). **e** Correlation coefficient graph revealing the correlations of EMP3 with common immunosuppressive factors in the TCGA_seq GBM dataset (*n* = 153). **f** Representative images of immunofluorescence (IF) staining for EMP3 and CD206 in different areas of serial sections from human GBM samples (*n* = 27). Scale bar = 50 μm. **g** Representative images of IF staining for EMP3 and PD-L1 in different areas of serial sections of human GBM samples (*n* = 27). Scale bar = 50 μm.**h** Representative images of IF staining for EMP3 and CD8α in different areas of serial sections from human GBM samples (*n* = 27). Scale bar = 50 μm. I. Representative images of IF staining for EMP3 and CD4 in different areas of serial sections of human GBM samples (*n* = 27). Scale bar = 50 μm. The mean ± S.D. is shown. Ns: nonsignificant, *p < 0.05, **p < 0.01, and ***p < 0.001

To determine the immunosuppressive role of EMP3 in GBM, we compared GBM transcriptional profiles from TCGA and CGGA datasets, together with signatures for GBM-mediated immunosuppressive gene sets [11, 18]. Single-sample GSEA (ssGSEA) analysis was conducted. The results revealed that *EMP3* was associated with immunosuppression (Fig. 1a-b, Supplementary Fig. 1A-B). By the CIBERSORT algorithm, the higher *EMP3* expression group exhibited higher percentages of M2 TAMs and CD4 memory resting T cells but lower percentages of CD8 T cells and CD4 memory activated T cells in the TCGA dataset (Fig. 1c). However, in the CGGA dataset, there was no significant difference between the high *EMP3* expression group and the low *EMP3* expression group (Supplementary Fig. 1C). We found that *EMP3* expression correlated with the expression of M2 TAM markers in both the TCGA and CGGA datasets (Fig. 1d, Supplementary Fig. 1D) and that *EMP3* was correlated with immunosuppressive factors of tumour cells and factors of exhausted cytotoxic T lymphocytes (Fig. 1e, Supplementary Fig. 1E). In human and mouse GBM tissues, we performed immunofluorescence (IF) staining to evaluate EMP3, M2 TAMs, and PD-L1. We observed elevated EMP3 in GBM areas with high expression of PD-L1 and abundant M2 TAM infiltration (Fig. 1f-g, Supplementary Fig. 1F-I). Moreover, we also evaluated CD4^+^ and CD8^+^ T cells in human GBM tissues and found that high expression of EMP3 resulted in a lack of CD4^+^ and CD8^+^ T cell infiltration (Fig. 1h-i, Supplementary Fig. 1J-K). These findings indicate that EMP3 is associated with immunosuppression in GBM.

### EMP3 induces M2 TAM polarization and recruitment in GBM

**Fig. 2:**
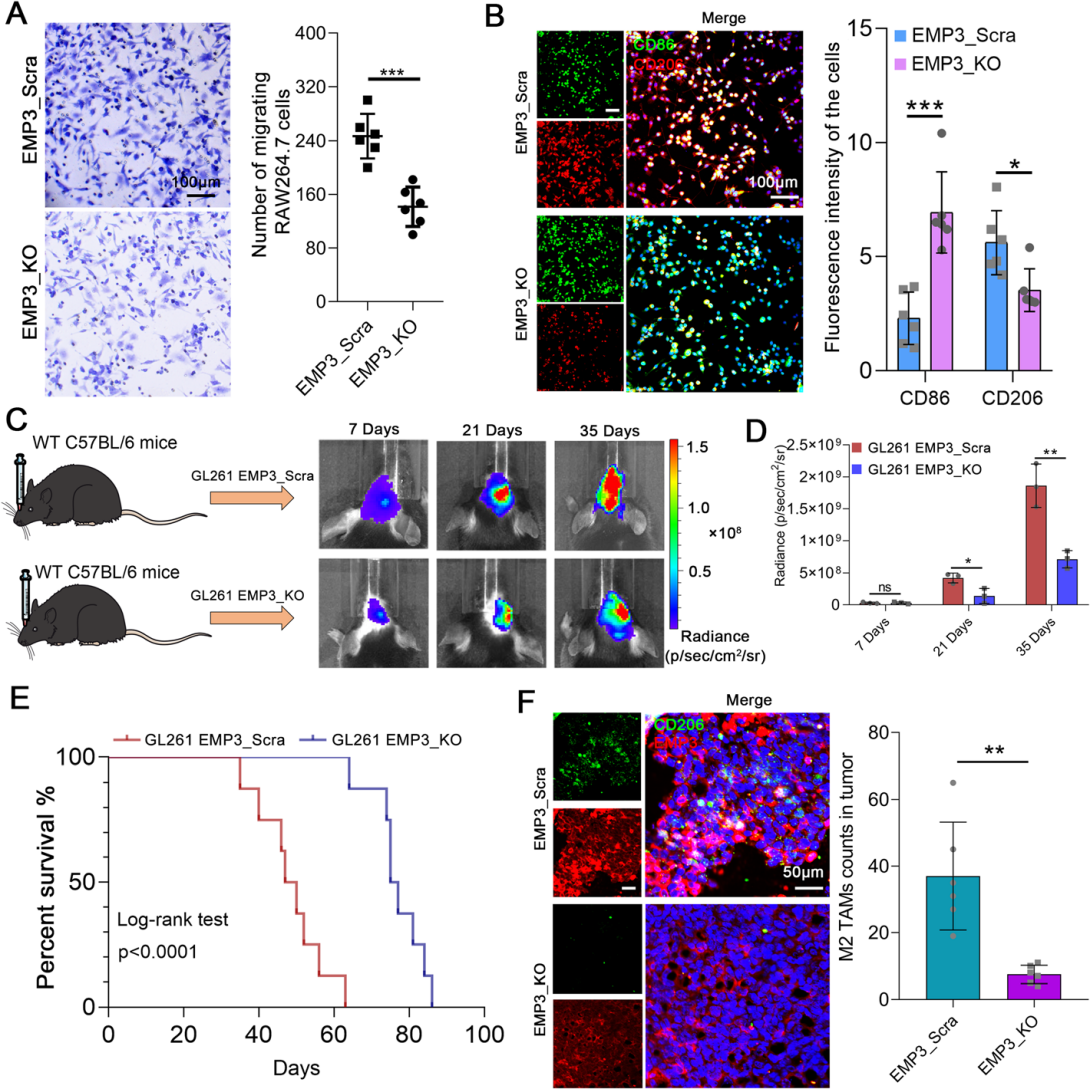
EMP3 could induce macrophage polarization. **a** Migration assays performed with RAW264.7 cells exposed to supernatants from EMP3_KO GL261 cells or EMP3_Scra GL261 cells. Scale bar = 100 μm. The histogram summarizes the number of migrating cells. Student’s t-test was performed. KO: Knockout; Scra: Scramble. **b** Representative images of IF staining for CD86 and CD206 in mouse BV-2 cells exposed to supernatants from EMP3_KO GL261 cells or EMP3_Scra GL261 cells. The histogram summarizes the fluorescence intensity of the cells. Student’s t-test was performed. Scale bar = 100 μm. KO: Knockout; Scra: Scramble. **c** Left: Schematic diagram of the orthotopic GBM model. Right: Bioluminescence images of BALB/c mice in the EMP3_KO and EMP3_Scra GL261 groups on days 14, 21 and 35. KO: Knockout; Scra: Scramble. **d** Quantification of the bioluminescence imaging signal intensities in C57BL/6 mice. Student’s t-test was performed.**e** Kaplan-Meier survival curves of mice bearing intracranial EMP3_KO or EMP3_Scra GL261 tumours. KO: Knockout; Scra: Scramble. The log-rank test was performed. **f** Representative images of IF staining for EMP3 and CD206 in mouse brain sections from the EMP3_KO and EMP3_Scra GL261 groups. The histogram summarizes the fluorescence intensity of the isolated brain tumour tissues (*n* = 3). KO: Knockout; Scra: Scramble. Student’s t-test was performed. Scale bar = 50 μm. The mean ± S.D. is shown. Ns: nonsignificant, *p < 0.05, **p < 0.01, and ***p < 0.001

M2 TAMs have been verified to have functions that promote GBM progression. We hypothesized that GBM-derived EMP3 is critical in the M2 polarization and recruitment of TAMs. Consistently, GSEA with gene sets featuring leukocyte migration genes showed that these genes were markedly overrepresented in GBM patients with high *EMP3* expression (Supplementary Fig. 2A). CRISPR/Cas9 was used to knock out EMP3 in GL261 cells (Supplementary Fig. 2B) and supernatants from GL261 cells promoted RAW264.7 cell migration (Fig. 2a). We observed reductions in M2 TAMs markers, such as ARG-1, MRC1 and IL-10, as well as an increase in the M1 marker TNF-α measured by qRT-PCR in RAW264.7 cells (Supplementary Fig. 2C–D). We found that RAW264.7 cells treated with supernatants from EMP3_KO GL261 cells produced less IL-10 but more TNF-α by using ELISA (Supplementary Fig. 2E). BV-2 cells incubated with supernatants from GL261 cells showed positive staining for both CD86 and CD206, indicating that M2 TAMs could be polarized by EMP3 (Fig. 2b). To further explore the effects of GBM-derived EMP3 on M2 TAM polarization *in vivo*, C57BL/6 mice were intracranially implanted with 4 × 10^5^ GL261 EMP3_KO or EMP3_Scra cells and monitored for tumour growth. We observed that knockout of EMP3 in GL261 cells suppressed tumour growth and prolonged mouse survival (Fig. 2c-e). The number of M2 TAMs infiltrating GBM tumours was reduced in the EMP3_KO group (Fig. 2f). These results reveal that GBM-derived EMP3 plays critical roles in promoting M2 TAM recruitment and polarization.

### EMP3 induces CCL2 and TGF-β1 secretion in GBM cells to promote M2 TAM recruitment and polarization

**Fig. 3:**
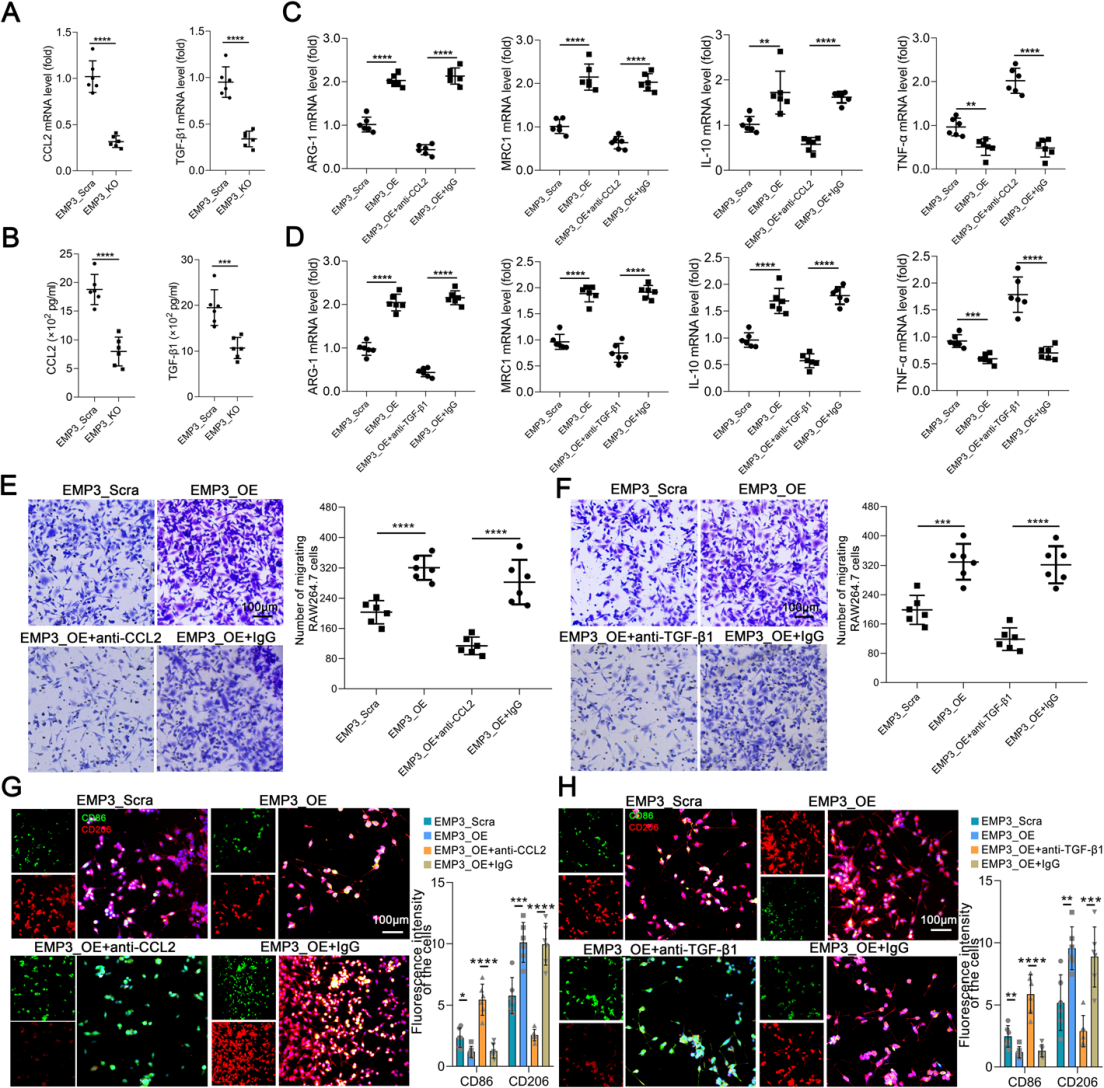
EMP3 induces CCL2 and TGF-β1 in GBM cells to promote M2 TAM recruitment and polarization. **a** CCL2 and TGF-β1 mRNA expression in EMP3_KO and EMP3_Scra GL261 cells was measured by qRT-PCR. The expression of these transcripts was normalized to that of β-actin. Student’s t-test was performed. KO: Knockout; Scra: Scramble. **b** The expression of CCL2 and TGF-β1 in EMP3_KO and EMP3_Scra GBM cell supernatants was measured by ELISA. Student’s t-test was performed. KO: Knockout; Scra: Scramble. **c**-**d** The ARG-1, MRC1, IL-10, and TNF-α mRNA expression in RAW264.7 cells exposed to supernatants from EMP3_OE, EMP3_Scra, EMP3_OE+anti-TGF-β1/anti-CCL2, or EMP3_OE+IgG GL261 cells for 48 hours was detected by qRT-PCR. The expression of these transcripts was normalized to that of β-actin. Student’s t-test was performed. OE: Overexpression; Scra: Scramble. **e**-**f** Migration assays performed with RAW264.7 cells exposed to supernatants from EMP3_OE, EMP3_Scra, EMP3_OE+anti-TGF-β1/anti-CCL2, or EMP3_OE+IgG GL261 cells for 48 hours. Scale bar = 100 μm. OE: Overexpression; Scra: Scramble. Student’s t-test was performed.**g**-**h** Representative images of IF staining for CD86 and CD206 in BV-2 cells exposed to supernatants from EMP3_OE, EMP3_Scra, EMP3_OE+anti-TGF-β1/anti-CCL2, or EMP3_OE+IgG GL261 cells for 48 hours. The histogram summarizes the relative fluorescence intensity of the cells. Scale bar = 100 μm. Student’s t-test was performed. KO: Knockout; Scra: scramble. The mean ± S.D. is shown. Ns: nonsignificant, *p < 0.05, **p < 0.01, and ***p < 0.001

Cytokines contribute to the infiltration of immune cells, including TAMs. M2 TAMs can be induced with CCL2 and TGF-β1 [19]. To illustrate the mechanism of EMP3-mediated recruitment and polarization of M2 TAMs in GBM, we found that EMP3_KO GL261 cells exhibited low mRNA expression of CCL2 and TGF-β1 (Fig. 3a). EMP3_KO GL261 cells produced less CCL2 and TGF-β1 than GL261 cells (Fig. 3b, Supplementary Fig. 3). We found that there was a reduction in the mRNA levels of M2 markers and an increase in the mRNA levels of an M1 marker after CCL2 and TGF-β1 were blocked in the EMP3 overexpression groups (Fig. 3c-d). Then, we further examined the roles of EMP3-induced CCL2 and TGF-β1 in GBM cells in promoting M2 TAM recruitment and polarization. We found that supernatants from GL261 cells treated with anti-CCL2 or anti-TGF-β1 monoclonal antibodies attenuated the promotive effects of EMP3 on monocyte migration and polarization (Fig. 3e-h). These findings suggest that EMP3 regulates CCL2 and TGF-β1 secretion in GBM cells to induce a change in M2 TAM recruitment and polarization.

### EMP3 inhibits T cell infiltration in GBM

**Fig. 4:**
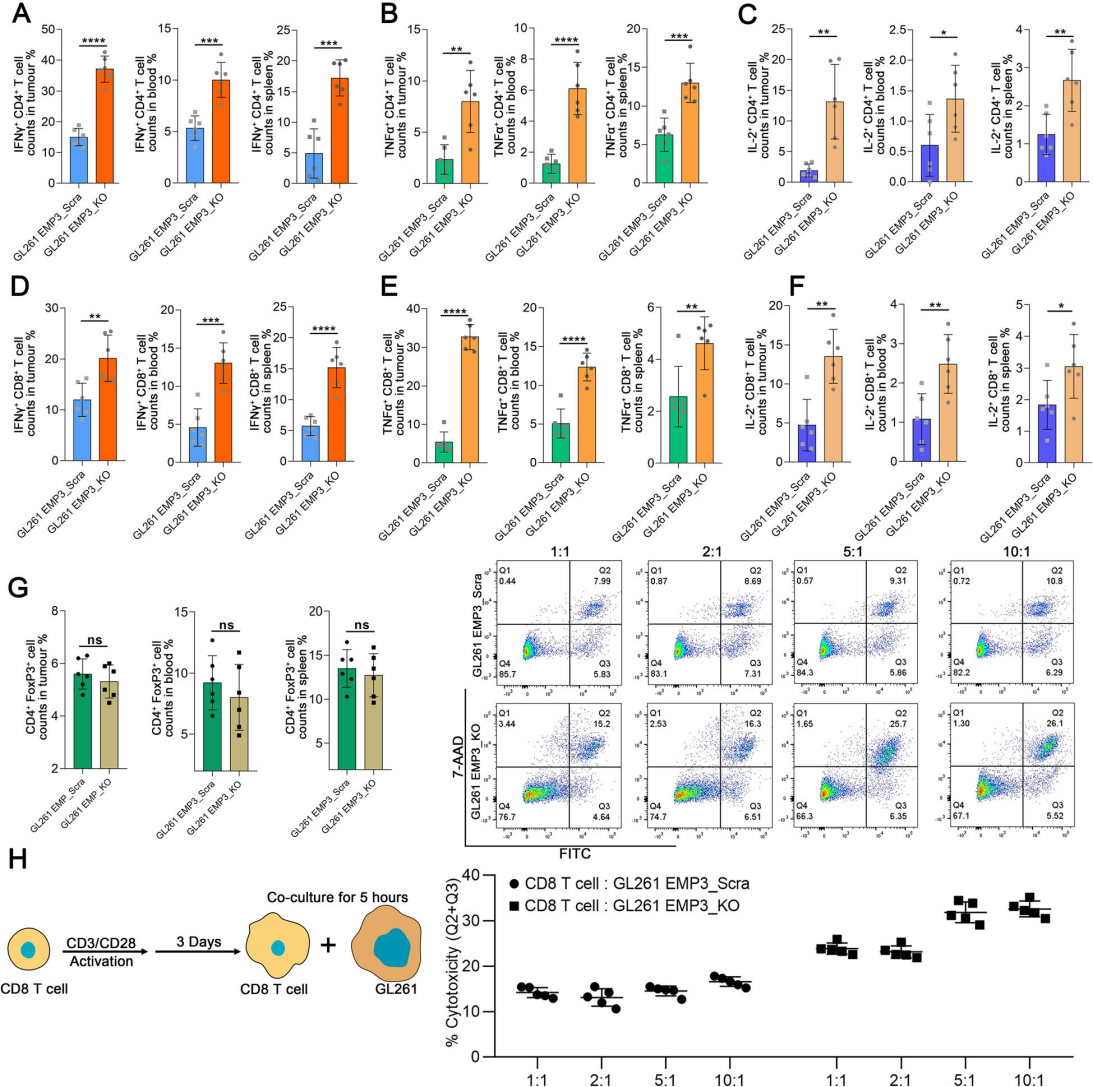
EMP3 drives T cell exclusion from the GBM microenvironment. **a**-**c** Percentages of IFN-γ^+^/TNF-α^+^/IL-2^+^ CD4^+^ T cells in mouse tumours, blood, and spleens from the EMP3_KO and EMP3_Scra GL261 groups. Student’s t-test was performed. **d**-**f** Percentages of IFN-γ^+^/TNF-α^+^/IL-2^+^CD8^+^ T cells in mouse tumours, blood, and spleens from the EMP3_KO and EMP3_Scra GL261 groups. Data were analysed using Student’s t-test. **g** Percentages of FoxP3^+^ CD4^+^ T cells in mouse tumours, blood, and spleens from the EMP3_KO and EMP3_Scra GL261 groups. Student’s t-test was performed.**h** CD8^+^ T cells were isolated from splenocytes from C57BL/6 mice and activated with anti-CD3/CD28 Dynabeads for 3 days. Activated CD8^+^ T cells were co-cultured with EMP3_KO or EMP3_Scra GL261 cells at ratios of 1:1, 2:1, 5:1, and 10:1. Cell apoptosis analysis was conducted with the Annexin V-FITC Apoptosis Detection Kit (BD Biosciences, USA) according to the manufacturer’s instructions. Student’s t-test was performed. The mean ± S.D. is shown. Ns: nonsignificant, *p < 0.05, **p < 0.01, and ***p < 0.001

Because EMP3 could regulate immunological effects, we detected T cell composition and functional changes to clarify the immune mechanisms responsible for the prolonged survival time of glioma-bearing mice with EMP3 deficiency. GBM-bearing mice were euthanized on day 14 after tumour implantation to isolate immune cells from the blood, spleen, and brain. We found that the frequencies of IFN-γ-, TNFα-, and IL-2-producing CD4^+^ T cells were increased in the brain tumour, spleen, and blood of EMP3_KO model mice (Fig. 4a-c). There were also increased frequencies of IFN-γ-, TNFα-, and IL-2-producing CD8^+^ T cells in the EMP3-deficient model mice (Fig. 4d-f). However, there were no significant reductions in the frequency of FoxP3^+^ Tregs in the tumour, blood, or spleen (Fig. 4g). Next, we determined whether there was increased CD8^+^ T cell-mediated killing activity against EMP3_KO GBM cells. At a T cell: GBM cell ratio of 5:1 or 10:1, there was an increase in murine CD8^+^ T cell cytotoxicity against EMP3_KO GL261 cells (Fig. 4h). Cumulatively, our data indicate that the elevation in T cell activity results from EMP3 deficiency in GBM cells and that EMP3 plays a critical role in suppressing T cell function.

### Loss of EMP3 promotes T cell infiltration via the CXCR3 system in GBM

**Fig. 5:**
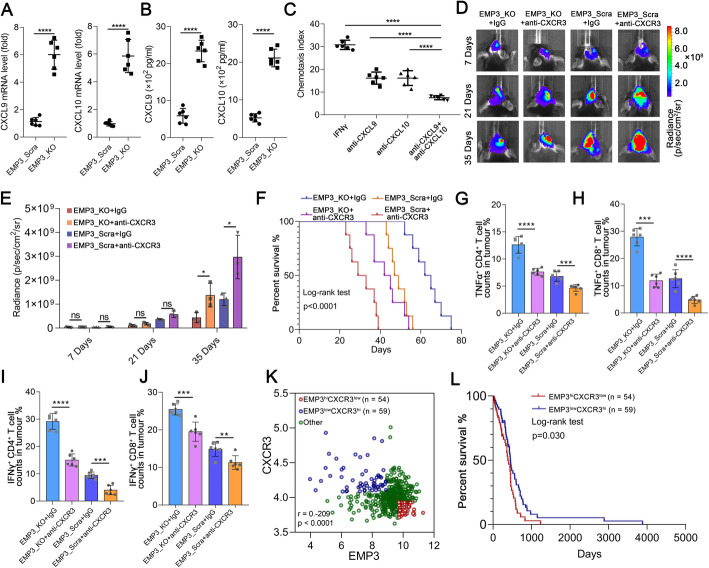
Loss of EMP3 in GBM cells promotes T cell infiltration via CXCL10 and CXCL9 in macrophages. **a**-**b** CXCL9 and CXCL10 mRNA and protein expression in RAW264.7 cells exposed to supernatants from EMP3_KO or EMP3_Scra GL261 cells for 48 hours was detected by qRT-PCR and ELISA. Student’s t-test was performed. KO: Knockout; Scra: Scramble. **c** Chemotaxis of T cells towards RAW264.7 cells treated with IFN-γ, anti-CXCL9 and/or CXCL10. Student’s t-test was performed.**d** Bioluminescent images of C57BL/6 mice treated with an anti-αCXCR3 antibody in the EMP3_KO and EMP3_Scra groups on days 14, 21 and 35. KO: Knockout; Scra: Scramble. **e** Quantification of the bioluminescence imaging signal intensities in C57BL/6 mice. Student’s t-test was performed. **f** Kaplan-Meier survival curves of mice bearing intracranial EMP3_KO or EMP3_Scra GL261 tumours treated with the anti-CXCR3 antibody or isotype IgG. KO: Knockout; Scra: Scramble. The log-rank test was performed. **g**-**h **Percentages of TNFα^+^ CD4^+^and TNFα^+^ CD8^+^ T cells in tumours from the EMP3_KO and EMP3_Scra GL261 groups treated with the anti-CXCR3 antibody or isotype IgG. Student’s t-test was performed. KO: Knockout; Scra: Scramble. **i**-**j** Percentages of IFN-γ^+^ CD4^+^ and IFN-γ^+^ CD8^+^T cells in tumours from the EMP3_KO and EMP3_Scra GL261 groups treated with the anti-CXCR3 antibody or isotype IgG. Student’s t-test was performed. KO: Knockout; Scra: Scramble.**k** Pearson correlation plots of the expression of EMP3 and CXCR3 in 470 GBM cases and the definition of 3 subgroups according to the expression patterns. **l** Kaplan-Meier survival analysis according to the EMP3-CXCR3 subgroups, as defined in (**k**). The log-rank test was performed. The mean ± S.D. is shown. Ns: nonsignificant, *p < 0.05, **p < 0.01, and ***p < 0.001

Macrophages can suppress infiltrating T cells in the TME [20]. Thus, we explored the mechanism by which EMP3-induced TAMs regulate T cell infiltration into the TME. Chemokines play a critical role in the infiltration of T cells [21]. We found that CXCL9 and CXCL10 mRNA and protein expression was increased in RAW264.7 cells incubated with conditioned media from the EMP3_KO groups (Fig. 5a-b). *CXCL9* and *CXCL10* expression was positively associated with both *CD4* and *CD8A* in the TCGA dataset (Supplement Fig. 4A-D). In addition, we found that *CXCL9* expression was positively correlated with *CD4* and *CD8A* in the CGGA dataset (Supplementary Fig. 4E-F). We found that *CXCL10* expression was not correlated with *CD4* but positively correlated with *CD8A* in the CGGA dataset (Supplementary Fig. 4G-H). Furthermore, *in vitro*, inhibition of CXCL9 and/or CXCL10 reduced T cell migration (Fig. 5c). CXCR3 and its ligands (CXCL9 and CXCL10) exquisitely control T cell trafficking and are essential for the differentiation, activation, and function of T cells [22, 23]. *In vivo*, an anti-CXCR3 antibody reversed the suppression of GBM growth seen in the EMP3_KO group but further promoted tumorigenesis in the EMP3_Scra group and led to poor survival (Fig. 5d-f). When we evaluated the percentages of IFN-γ-, TNF-α-, and IL-2-producing CD4^+^ and CD8^+^ T cells within GBM tumours, we noticed that CXCR3 blockade in the EMP3_KO group reduced CD8^+^ and CD4^+^ T cell infiltration into the brain tumour, blood, and spleen (Fig. 5g-j, Supplementary Fig. 5A-G). Given the above observations, we next asked whether co-expression of EMP3 and CXCR3 has a prognostic impact in GBM. In the TCGA dataset, we found a negative association between *CXCR3* and *EMP3*, and patients with low *EMP3* expression and high *CXCR3* expression (*EMP3*^low^*CXCR3*^hi^) survived longer (Fig. 5k-l). Collectively, our findings revealed that EMP3-induced T cell exclusion from the GBM microenvironment is mediated through the CXCR3 system.

### Knockout of EMP3 and PD-1 blockade have synergistic anti-GBM effects

**Fig. 6:**
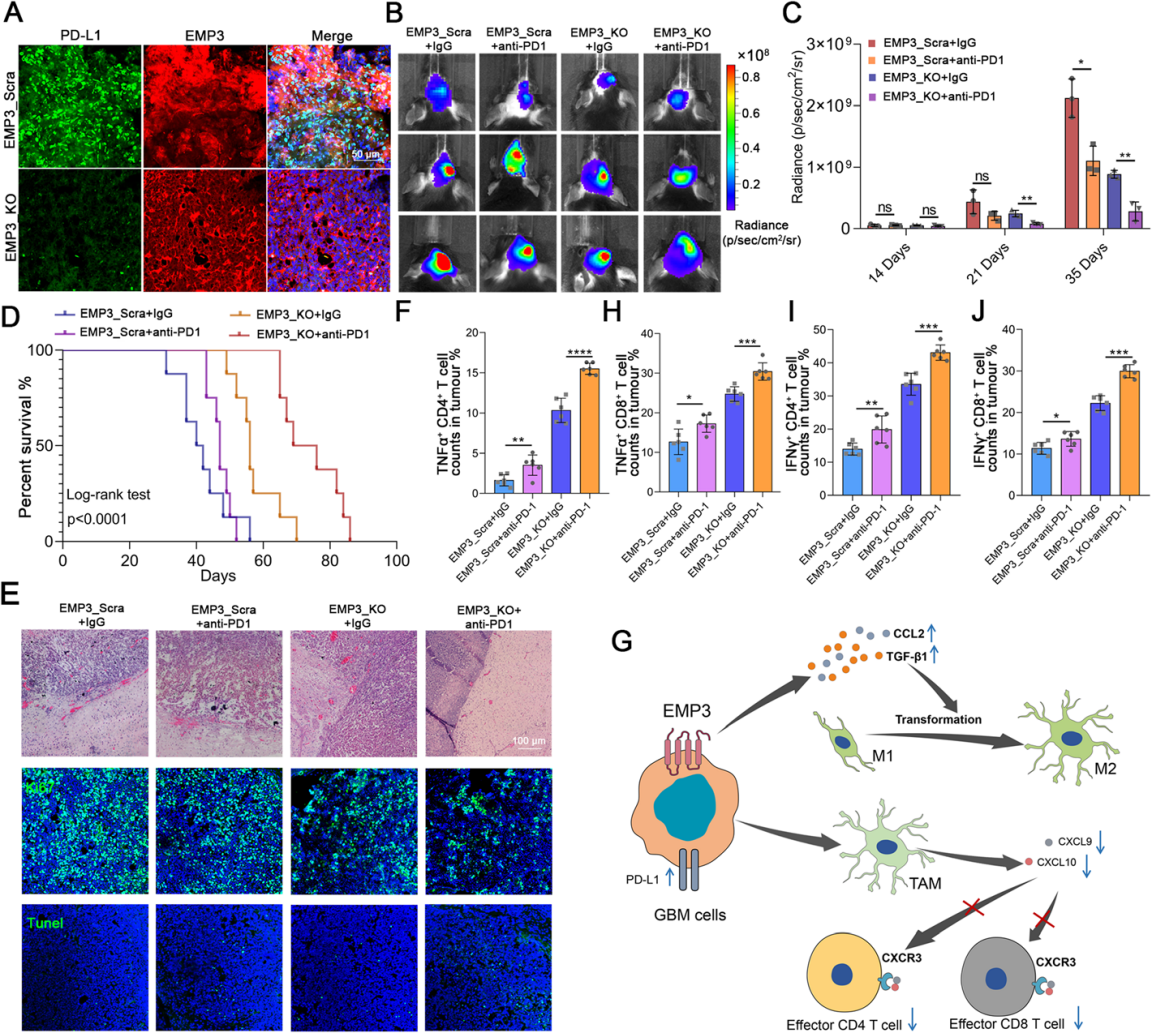
Knockout of EMP3 and immune checkpoint blockade have synergistic antitumorigenic effects. **a** Representative images of IF staining for EMP3 and PD-L1 in mouse brain tissues from the EMP3_KO and EMP3_Scra GL261 groups. Scale bar = 50 μm. KO: Knockout; Scra: Scramble. **b** Bioluminescence images of EMP3_KO and EMP3_Scra C57BL/6 mice treated with isotype IgG or PD1 blockade. KO: Knockout; Scra: Scramble. **c** Quantification of the bioluminescence imaging signal intensities in C57BL/6 mice. Student’s t-test was performed. **d** Kaplan-Meier survival curves of mice bearing intracranial EMP3_KO or EMP3_Scra GL261 tumours treated with an anti-PD1 antibody or isotype IgG. KO: Knockout; Scra: Scramble. The log-rank test was performed. **e** Tumour sections from the EMP3_KO and EMP3_Scra GL261 groups treated with the anti-PD1 antibody or isotype IgG were used for a TUNEL assay, IF staining and H&E analysis. Upper: Fluorescence images of TUNEL staining in tumours. Middle: Representative images of IF staining for Ki67 in tumours. Lower: H&E staining of tumour sections. Scale bar = 100 μm.**f**-**h** Percentages of TNFα^+^ CD4^+^ and TNFα^+^ CD8^+^T cells in tumours from the EMP3_KO and EMP3_Scra GL261 groups treated with the anti-PD1 antibody or isotype IgG. Student’s t-test was performed. KO: Knockout; Scra: Scramble. **i**-**j** Percentages of IFN-γ^+^ CD4^+^and IFN-γ^+^ CD8^+^ T cells in tumours from the EMP3_KO and EMP3_Scra GL261 groups treated with the anti-PD1 antibody or isotype IgG. Student’s t-test was performed. KO: Knockout; Scra: Scramble. **g** Schematic showing that EMP3 mediates TAM infiltration to suppress T cell infiltration. The mean ± S.D. is shown. Ns: nonsignificant, *p < 0.05, **p < 0.01, and ***p < 0.001

In GBM, most intratumoural T cells are exhausted with loss of effector function; hence, these T cells do not appear to be responsive to immune checkpoint blockade therapy. TAMs can inhibit antigen presentation and suppress the migration of antigen-presenting cells to regional lymph nodes [1, 24]. We performed immunostaining for PD-L1 in mouse GBM tissues to assess the effect of EMP3. We found that PD-L1 was decreased in EMP3_KO tumours (Fig. 6a), indicating that EMP3 could induce the immunosuppressive ligand PD-L1 in GBM. Therefore, we explored whether EMP3 deficiency can improve the outcomes of mice receiving anti-PD1 therapy. We found that PD1 blockade alone had relatively little effect on tumour growth. However, knockout of EMP3 combined with anti-PD1 therapy resulted in an improvement in tumour growth delay (Fig. 6b-c). Overall survival was prolonged in the EMP3_KO plus anti-PD1 treatment group (Fig. 6d). Histological, IF, and TUNEL assays confirmed the decreased invasiveness and proliferation but increased apoptosis of tumours in the EMP3_KO plus anti-PD-1 treatment group (Fig. 6e). We also evaluated the percentages of CD8^+^ and CD4^+^ T cells within the tumour, blood, and spleen and found that anti-PD-1 treatment of EMP3_KO tumour-bearing mice increased IFN-γ-, TNF-α- and IL-2-producing CD8^+^ and CD4^+^ T cell infiltration into the brain tumour, blood, and spleen (Fig. 6f-j, Supplementary Fig. 6A-G). Our findings suggest that EMP3 inhibition enhances the PD1 blockade response in GBM.

## Discussion

Immunosuppression has been systemically demonstrated, as evidenced by impaired cellular immunity in GBM patients and murine GBM models [25]. While immunotherapy-based approaches have been widely attempted, most clinical trials involving these modalities have failed to report significant outcomes [26, 27]. Indeed, GBMs have a small quantity of infiltrating T cells and harbour abundant M2 TAMs [28]. M2 TAMs can promote tumour cell progression directly by activating angiogenesis, maintaining tumour cell stemness, and inducing drug resistance and indirectly by facilitating dysfunctions in the immune system within the TME, ultimately resulting in immune evasion by cancer cells [2, 3]. M2 TAMs are known to potentiate immunosuppression in GBM and may contribute to the failure of immunotherapies for gliomas [1]. Our data propose a unifying mechanism of EMP3 underlying the crosstalk between GBM cells and the immune system. In GBM, EMP3 expression is upregulated to promote the progression of brain tumours, and high expression of EMP3 is associated with a poor prognosis [9, 12]. From our in silico analyses of the TCGA and CGGA datasets, EMP3 was found to be involved in immunosuppression in GBM. Compared to the CGGA dataset, the TCGA dataset showed an increased percentage of M2 TAMs but a decreased percentage of CD8 T cells in the high EMP3 expression group. The main reason for the inconsistency in predicting the relationship between EMP3 and immune cells is that the number of patients in the CGGA dataset (*n* = 96) is smaller than that in the TCGA dataset (*n* = 521). We found that knockout of EMP3 repressed tumorigenesis and produced a survival advantage in mice. A previous study reported that EMP3 could interact with TGF-β receptor type 2 (TGFBR2) upon TGF-β stimulation in GBM cells. Consequently, the EMP3-TGFBR2 interaction activates TGF-β/Smad2/3 signalling and positively impacts TGF-β-stimulated gene expression and cell proliferation *in vitro* and *in vivo* [12]. Our findings provide further evidence that EMP3 deficiency in GBM is critical for inhibiting the polarization and recruitment of M2 TAMs by reducing the production of CCL2 and TGF-β1 by GBM cells. It has also been reported that knockdown of EMP3 results in reduced levels of p-AKT, p-ERK and p-EGFR and attenuated cell proliferation, indicating that EMP3 is involved in the regulation of receptor tyrosine kinase-mediated mitogenic signalling [29]. In GBM patients, decreases in CD4^+^ and CD8^+^ T cell numbers within the tumour and in the circulation are common, and the T cell population is prone to exhaustion [30, 31]. It has been reported that osteopontin in GBM cells results in an increase in M2 TAMs and reduces T cell infiltration in tumour areas [32]. L19-mIL12, comprising murine IL12 fused to the L19 antibody, has a strong effect on tumour-infiltrating immune cells, increasing the infiltration and activation of CD4 and CD8 T cells in mouse glioma models [33]. In our current research, knockout of EMP3 enhanced the induction of T cell infiltration and secretion of TNF-α, INF-γ, and IL-2 by CD4^+^ and CD8^+^ T cells. Elevated expression of *CXCL9* and *CXCL10* was positively associated with CD4 and CD8 T cell markers in the TCGA and CGGA datasets. Moreover, we observed that EMP3 affected macrophages, causing inhibition of antitumour immunity via suppression of CD4^+^ and CD8^+^ T cell infiltration into GBM tumours and downregulation of CXCL9 and CXCL10 production by macrophages. These findings support the view that EMP3 plays a critical role in suppressing TAM-derived CXCL9- and CXCL10-mediated CD4^+^ and CD8^+^ T cell infiltration into the TME to promote tumour progression. CXCR3, a chemokine receptor for the interferon-inducible chemokines CXCL9 and CXCL10, is highly expressed on activated T cells and plays pivotal roles in the spatial distribution, migration, and function of T cells [34]. *In vivo*, blockade of CXCR3 abrogated the suppression of GBM growth observed in the EMP3_KO group but promoted tumorigenesis in the EMP3_Scra group and led to significantly poorer survival. The patients in the *EMP3*^low^*CXCR3*^hi^ group survived longer. Functional immune assays also demonstrated that inhibition of CXCR3 suppressed T cell infiltration. To some extent, these results suggest that EMP3 and CXCR3 have an antagonistic effect on glioma.

The increased T cell populations may be due to increased infiltration or reduced T cell death within tumours. Exhaustion has been shown to facilitate T cell apoptosis via the PD-1/PD-L1 axis and therefore may contribute to the loss of T cells at the tumour site [17, 35]. Clinical trials of immune checkpoint blockade therapies, predominantly targeting PD-1 or PD-L1, have been ongoing in GBM, although initial results have been disappointing [36]. In our present study, anti-PD1 therapy alone showed minimal improvement in EMP3_Scra tumours. However, in EMP3_KO tumours, the treatment produced a greater antitumour response. In addition, increased populations of both CD4^+^ and CD8^+^ T cells within GBM tumours were observed. In glioma, PD-1 expression on the surface of T cells is one of the hallmarks of T cell exhaustion. PD-1^+^ T cells have a narrower range of cognate antigen targets than PD-1^−^ T cells, accounting for the immune responses to PD-1 blockade therapies [37]. Increased expression of PD-L1 suppresses CD8^+^ T cell activation and acquisition of an effector phenotype. Reduced PD-L1 expression accompanied by PD-1-targeted immunotherapy diminishes tumour growth and increases survival dependent on CD8^+^ T cells [38–40]. In our GBM specimens, we found that the expression of EMP3 was positively associated with PD-L1. *In vivo*, we found that knockout of EMP3 downregulated PD-L1, which might explain the increased cell killing demonstrated by CD8^+^ T cells against EMP3_KO cells. Moreover, to strengthen PD-1 inhibitor efficacy, ablating M2 TAMs has been shown to enhance anti-PD-1 immunotherapy in different GBM subtypes [41, 42]. In our present study, it was showed that EMP3 could induce M2 TAM polarization and recruitment in GBM. The above findings indicate that EMP3 has a synergistic effect with PD-1 in glioma.

## Conclusions

To summarize, we uncovered the important roles of EMP3 in mediating M2 TAM infiltration and suppressing intratumoural T cell recruitment (Fig. 6g). In addition, EMP3 deficiency decreased PD-L1 expression, augmented anti-PD-1 treatment, and produced a survival advantage in glioma-bearing mice. Our findings indicate that the combination of EMP3 inhibition and PD-1 blockade is a promising therapy for GBM patients.

## Supplementary Information

**Additional file 1.**

## Data Availability

Not applicable.

## Abbreviations

GBM: Glioblastoma
TAMs: Tumour-associated macrophages
EMP3: Epithelial membrane protein 3
TME: Tumour microenvironment
ssGSEA: Single-sample GSEA
TCGA: The Cancer Genome Atlas
H&E: Haematoxylin and eosin

## References

[1] Jackson CM, Choi J, Lim M (2019). Mechanisms of immunotherapy resistance: lessons from glioblastoma. Nat Immunol.

[2] Biswas SK, Mantovani A (2010). Macrophage plasticity and interaction with lymphocyte subsets: cancer as a paradigm. Nat Immunol.

[3] Qian BZ, Pollard JW (2010). Macrophage diversity enhances tumor progression and metastasis. Cell.

[4] Kmiecik J, Poli A, Brons NH, Waha A, Eide GE, Enger PO, Zimmer J, Chekenya M (2013). Elevated CD3 + and CD8 + tumor-infiltrating immune cells correlate with prolonged survival in glioblastoma patients despite integrated immunosuppressive mechanisms in the tumor microenvironment and at the systemic level. J Neuroimmunol.

[5] Raychaudhuri B, Rayman P, Huang P, Grabowski M, Hambardzumyan D, Finke JH, Vogelbaum MA (2015). Myeloid derived suppressor cell infiltration of murine and human gliomas is associated with reduction of tumor infiltrating lymphocytes. J Neurooncol.

[6] Topalian SL, Drake CG, Pardoll DM (2015). Immune checkpoint blockade: a common denominator approach to cancer therapy. Cancer Cell.

[7] Kusumoto Y, Okuyama H, Shibata T, Konno K, Takemoto Y, Maekawa D, Kononaga T, Ishii T, Akashi-Takamura S, Saitoh SI, et al. Epithelial membrane protein 3 (Emp3) downregulates induction and function of cytotoxic T lymphocytes by macrophages via TNF-alpha production. Cell Immunol. 2018;324:33–41. 10.1016/j.cellimm.2017.12.001 29269102

[8] Bhat KPL, Balasubramaniyan V, Vaillant B, Ezhilarasan R, Hummelink K, Hollingsworth F, Wani K, Heathcock L, James JD, Goodman LD (2013). Mesenchymal differentiation mediated by NF-kappaB promotes radiation resistance in glioblastoma. Cancer Cell.

[9] Ernst A, Hofmann S, Ahmadi R, Becker N, Korshunov A, Engel F, Hartmann C, Felsberg J, Sabel M, Peterziel H (2009). Genomic and expression profiling of glioblastoma stem cell-like spheroid cultures identifies novel tumor-relevant genes associated with survival. Clin Cancer Res.

[10] Gao YF, Zhu T, Mao CX, Liu ZX, Wang ZB, Mao XY, Li L, Yin JY, Zhou HH, Liu ZQ. PPIC, EMP3 and CHI3L1 Are Novel Prognostic Markers for High Grade Glioma. Int J Mol Sci. 2016,17:1808. 10.3390/ijms17111808 PMC5133809 27801851

[11] Chen Q, Han B, Meng X, Duan C, Yang C, Wu Z, Magafurov D, Zhao S, Safin S, Jiang C, Cai J (2019). Immunogenomic analysis reveals LGALS1 contributes to the immune heterogeneity and immunosuppression in glioma. Int J Cancer.

[12] Jun F, Hong J, Liu Q, Guo Y, Liao Y, Huang J, Wen S, Shen L (2017). Epithelial membrane protein 3 regulates TGF-beta signaling activation in CD44-high glioblastoma. Oncotarget.

[13] Subramanian A, Tamayo P, Mootha VK, Mukherjee S, Ebert BL, Gillette MA, Paulovich A, Pomeroy SL, Golub TR, Lander ES, Mesirov JP (2005). Gene set enrichment analysis: a knowledge-based approach for interpreting genome-wide expression profiles. Proc Natl Acad Sci U S A.

[14] Barbie DA, Tamayo P, Boehm JS, Kim SY, Moody SE, Dunn IF, Schinzel AC, Sandy P, Meylan E, Scholl C (2009). Systematic RNA interference reveals that oncogenic KRAS-driven cancers require TBK1. Nature.

[15] Wu P, Cai J, Chen Q, Han B, Meng X, Li Y, Li Z, Wang R, Lin L, Duan C (2019). Lnc-TALC promotes O(6)-methylguanine-DNA methyltransferase expression via regulating the c-Met pathway by competitively binding with miR-20b-3p. Nat Commun.

[16] Petty AJ, Li A, Wang X, Dai R, Heyman B, Hsu D, Huang X, Yang Y (2019). Hedgehog signaling promotes tumor-associated macrophage polarization to suppress intratumoral CD8 + T cell recruitment. J Clin Invest.

[17] Wu P, Geng B, Chen Q, Zhao E, Liu J, Sun C, Zha C, Shao Y, You B, Zhang W, et al: Tumor cell-derived TGFbeta1 Attenuates Antitumor Immune Activity of T cells via Regulation of PD-1 mRNA. Cancer Immunol Res. 2020;8:1470-1484. 10.1158/2326-6066.CIR-20-0113 32999004

[18] Cai J, Chen Q, Cui Y, Dong J, Chen M, Wu P, Jiang C (2018). Immune heterogeneity and clinicopathologic characterization of IGFBP2 in 2447 glioma samples. Oncoimmunology.

[19] An Z, Knobbe-Thomsen CB, Wan X, Fan QW, Reifenberger G, Weiss WA (2018). EGFR Cooperates with EGFRvIII to Recruit Macrophages in Glioblastoma. Cancer Res.

[20] Quintana E, Schulze CJ, Myers DR, Choy TJ, Mordec K, Wildes D, Shifrin NT, Belwafa A, Koltun ES, Gill AL (2020). Allosteric Inhibition of SHP2 Stimulates Antitumor Immunity by Transforming the Immunosuppressive Environment. Cancer Res.

[21] Dangaj D, Bruand M, Grimm AJ, Ronet C, Barras D, Duttagupta PA, Lanitis E, Duraiswamy J, Tanyi JL, Benencia F (2019). Cooperation between Constitutive and Inducible Chemokines Enables T Cell Engraftment and Immune Attack in Solid Tumors. Cancer Cell.

[22] Croudace JE, Inman CF, Abbotts BE, Nagra S, Nunnick J, Mahendra P, Craddock C, Malladi R, Moss PA (2012). Chemokine-mediated tissue recruitment of CXCR3 + CD4 + T cells plays a major role in the pathogenesis of chronic GVHD. Blood.

[23] Abron JD, Singh NP, Murphy AE, Mishra MK, Price RL, Nagarkatti M, Nagarkatti PS, Singh UP (2018). Differential role of CXCR3 in inflammation and colorectal cancer. Oncotarget.

[24] Wainwright DA, Chang AL, Dey M, Balyasnikova IV, Kim CK, Tobias A, Cheng Y, Kim JW, Qiao J, Zhang L (2014). Durable therapeutic efficacy utilizing combinatorial blockade against IDO, CTLA-4, and PD-L1 in mice with brain tumors. Clin Cancer Res.

[25] Lim M, Xia Y, Bettegowda C, Weller M (2018). Current state of immunotherapy for glioblastoma. Nat Rev Clin Oncol.

[26] Wen PY, Weller M, Lee EQ, Alexander BM, Barnholtz-Sloan JS, Barthel FP, Batchelor TT, Bindra RS, Chang SM, Chiocca EA (2020). Glioblastoma in adults: a Society for Neuro-Oncology (SNO) and European Society of Neuro-Oncology (EANO) consensus review on current management and future directions. Neuro Oncol.

[27] Mohme M, Neidert MC, Regli L, Weller M, Martin R (2014). Immunological challenges for peptide-based immunotherapy in glioblastoma. Cancer Treat Rev.

[28] Chongsathidkiet P, Jackson C, Koyama S, Loebel F, Cui X, Farber SH, Woroniecka K, Elsamadicy AA, Dechant CA, Kemeny HR (2018). Sequestration of T cells in bone marrow in the setting of glioblastoma and other intracranial tumors. Nat Med.

[29] Christians A, Poisel E, Hartmann C, von Deimling A, Pusch S (2019). Characterization of the epithelial membrane protein 3 interaction network reveals a potential functional link to mitogenic signal transduction regulation. Int J Cancer.

[30] Friebel E, Kapolou K, Unger S, Nunez NG, Utz S, Rushing EJ, Regli L, Weller M, Greter M, Tugues S, et al: Single-Cell Mapping of Human Brain Cancer Reveals Tumor-Specific Instruction of Tissue-Invading Leukocytes. Cell. 2020,181:1626–42 e1620. 10.1016/j.cell.2020.04.055 32470397

[31] Klemm F, Maas RR, Bowman RL, Kornete M, Soukup K, Nassiri S, Brouland JP, Iacobuzio-Donahue CA, Brennan C, Tabar V, et al: Interrogation of the Microenvironmental Landscape in Brain Tumors Reveals Disease-Specific Alterations of Immune Cells. Cell. 2020,181:1643–60 e1617. 10.1016/j.cell.2020.05.007 PMC8558904 32470396

[32] Wei J, Marisetty A, Schrand B, Gabrusiewicz K, Hashimoto Y, Ott M, Grami Z, Kong LY, Ling X, Caruso H (2019). Osteopontin mediates glioblastoma-associated macrophage infiltration and is a potential therapeutic target. J Clin Invest.

[33] Weiss T, Puca E, Silginer M, Hemmerle T, Pazahr S, Bink A, Weller M, Neri D, Roth P. Immunocytokines are a promising immunotherapeutic approach against glioblastoma. Sci Transl Med. 2020,12:eabb2311. 10.1126/scitranslmed.abb2311 33028706

[34] Chow MT, Ozga AJ, Servis RL, Frederick DT, Lo JA, Fisher DE, Freeman GJ, Boland GM, Luster AD. Intratumoral Activity of the CXCR3 Chemokine System Is Required for the Efficacy of Anti-PD-1 Therapy. Immunity. 2019,50:1498–512 e1495. 10.1016/j.immuni.2019.04.010 PMC6527362 31097342

[35] Meng X, Liu X, Guo X, Jiang S, Chen T, Hu Z, Liu H, Bai Y, Xue M, Hu R (2018). FBXO38 mediates PD-1 ubiquitination and regulates anti-tumour immunity of T cells. Nature.

[36] Tan AC, Ashley DM, Lopez GY, Malinzak M, Friedman HS, Khasraw M (2020). Management of glioblastoma: State of the art and future directions. CA Cancer J Clin.

[37] Davidson TB, Lee A, Hsu M, Sedighim S, Orpilla J, Treger J, Mastall M, Roesch S, Rapp C, Galvez M (2019). Expression of PD-1 by T Cells in Malignant Glioma Patients Reflects Exhaustion and Activation. Clin Cancer Res.

[38] Lamano JB, Lamano JB, Li YD, DiDomenico JD, Choy W, Veliceasa D, Oyon DE, Fakurnejad S, Ampie L, Kesavabhotla K (2019). Glioblastoma-Derived IL6 Induces Immunosuppressive Peripheral Myeloid Cell PD-L1 and Promotes Tumor Growth. Clin Cancer Res.

[39] Yamaguchi I, Nakajima K, Shono K, Mizobuchi Y, Fujihara T, Shikata E, Yamaguchi T, Kitazato K, Sampetrean O, Saya H, Takagi Y (2020). Downregulation of PD-L1 via FKBP5 by celecoxib augments antitumor effects of PD-1 blockade in a malignant glioma model. Neurooncol Adv.

[40] Ricklefs FL, Alayo Q, Krenzlin H, Mahmoud AB, Speranza MC, Nakashima H, Hayes JL, Lee K, Balaj L, Passaro C (2018). Immune evasion mediated by PD-L1 on glioblastoma-derived extracellular vesicles. Sci Adv.

[41] Cui X, Ma C, Vasudevaraja V, Serrano J, Tong J, Peng Y, Delorenzo M, Shen G, Frenster J, Morales RT, et al: Dissecting the immunosuppressive tumor microenvironments in Glioblastoma-on-a-Chip for optimized PD-1 immunotherapy. Elife. 2020,9:e52253. 10.7554/eLife.52253 PMC7556869 32909947

[42] Van Woensel M, Mathivet T, Wauthoz N, Rosiere R, Garg AD, Agostinis P, Mathieu V, Kiss R, Lefranc F, Boon L (2017). Sensitization of glioblastoma tumor micro-environment to chemo- and immunotherapy by Galectin-1 intranasal knock-down strategy. Sci Rep.

