# Supplementary material for: EMP3 mediates glioblastoma‐associated macrophage infiltration to drive T cell exclusion
Source: J Exp Clin Cancer Res. 2021 May 8;40:160. doi: 10.1186/s13046-021-01954-2 (PMC8106853; doi:10.1186/s13046-021-01954-2)
Supplement: Supplementary file 1 — Additional file 1. [file 13046_2021_1954_MOESM1_ESM.doc]

**Supplementary Figure Legends**

**
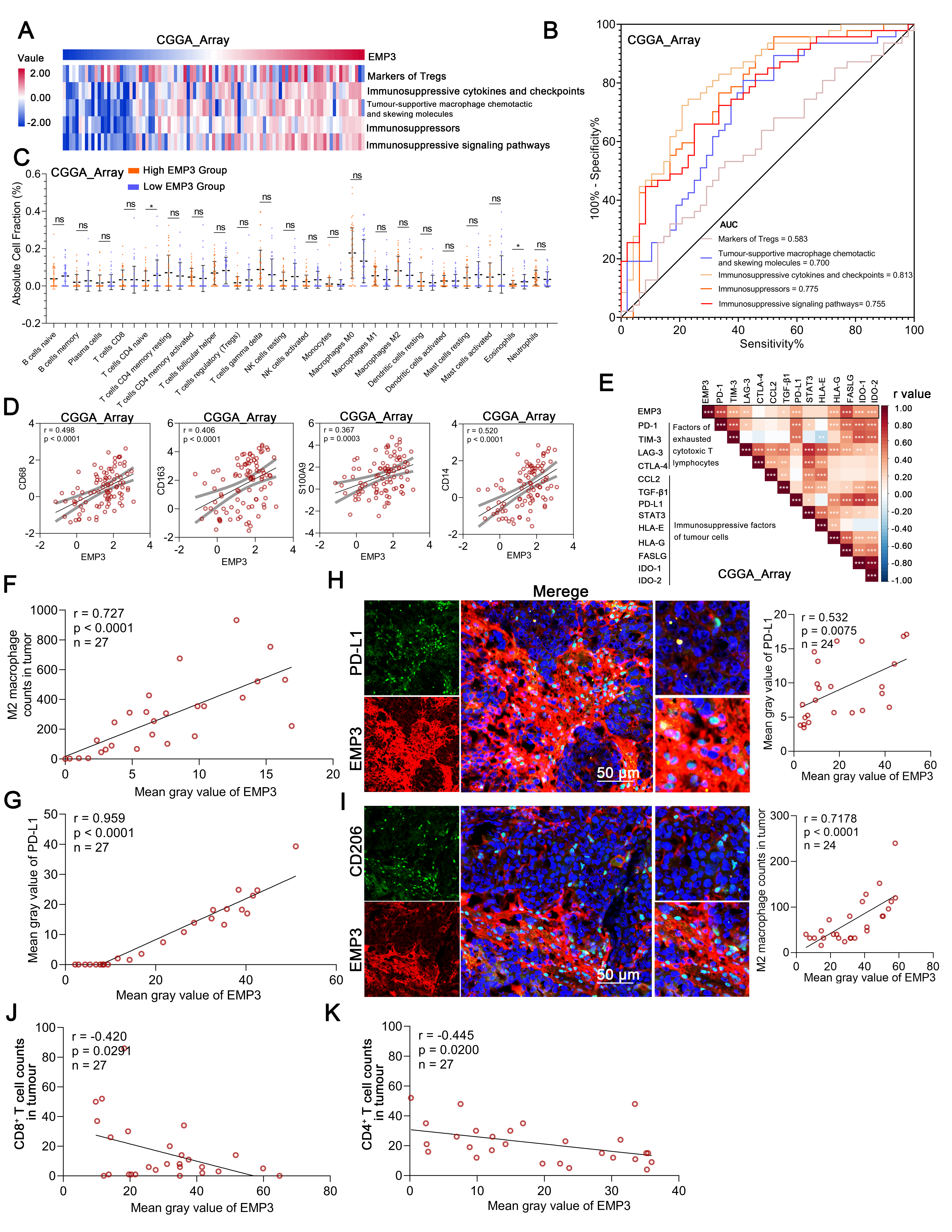
**

**Supplementary Figure 1. EMP3 is associated with immunosuppression in GBM.**

A. Pearson correlation analysis of EMP3 expression with GBM-associated immunosuppressive metagenes in the CGGA_array GBM dataset (n = 94).

B. ROC curves indicated that high EMP3 was involved in immunosuppression in the CGGA_array GBM dataset (n = 94).

C. Immune cell fractions were estimated using CIBERSORT, and the differences between the cell fractions of the high and low EMP3 groups in the CGGA_array GBM dataset were calculated using a t-test (n = 94).

D. Pearson correlation plots of the expression of EMP3 with M2 TAM signature markers (CD68, CD163, S100A9, and CD14) in the CGGA_array GBM dataset (n = 94).

E. Correlation coefficient graph revealing the correlations of EMP3 with common immunosuppressive factors in the CGGA_array GBM dataset (n = 94).

F. Pearson correlation plots of the expression of EMP3 and PD-L1 in GBM samples (n = 27).

G. Pearson correlation plots of the expression of EMP3 and M2 TAMs in GBM samples (n = 27).

H. Representative images of IF staining for EMP3 and PD-L1 in different areas of serial sections from mouse brain samples (n = 24). Scale bar = 50 μm. Pearson correlation plots of the expression of EMP3 and M2 TAMs in mouse brain tumour samples.

I. Representative images of IF staining for EMP3 and CD206 in different areas of serial sections from mouse brain tumour samples (n = 24). Scale bar = 50 μm. Pearson correlation plots of the expression of EMP3 and PD-L1 in mouse brain tumour samples.

J. Pearson correlation plots of the expression of EMP3 and CD8^+^ T cells in GBM samples (n = 27).

K. Pearson correlation plots of the expression of EMP3 and CD4^+^ T cells in GBM samples (n = 27).

The mean ± S.D. is shown. Ns: nonsignificant, *p < 0.05, **p < 0.01, and ***p < 0.001.


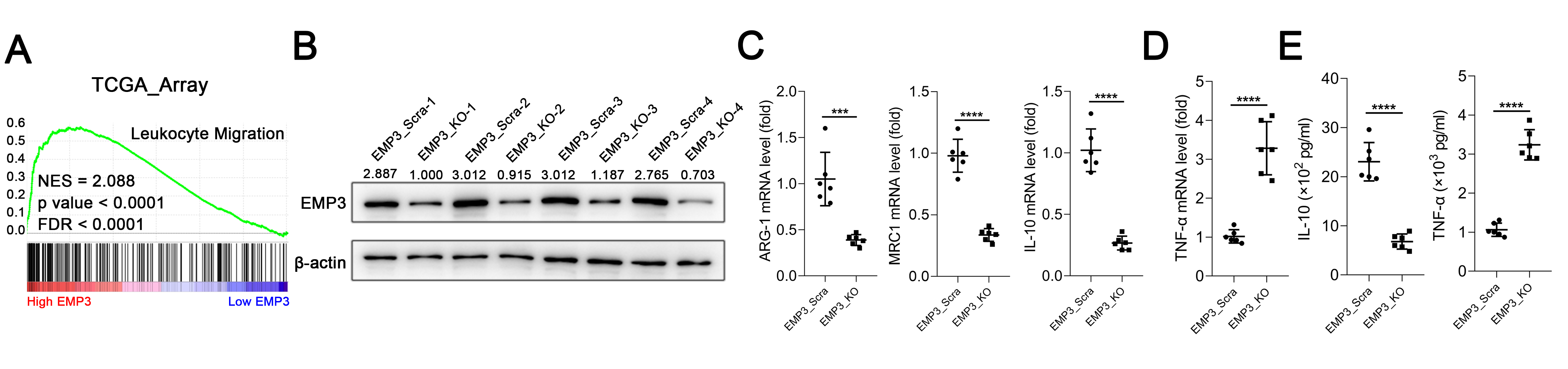
**Supplementary Figure 2. Knockout of EMP3 repressed M2 macrophage signature markers in GBM.**

A. Enrichment analysis of gene signatures of leukocyte migration between high and low EMP3 groups in the TCGA dataset.

B. Western blot analysis of EMP3 in GL261 cells infected with lentiviruses containing sgRNA targeting EMP3 for 48 hours.

C-D. qRT-PCR analysis of ARG-1, MRC1, IL-10, and TNF-α mRNA expression in RAW264.7 cells exposed to supernatants from EMP3_KO or EMP3_Scra GL261 cells. The expression of these transcripts was normalized to that of β-actin. KO: Knockout; Scra: Scramble. Student’s t-test was performed.

E. IL-10 and TNF-α protein expression in RAW264.7 cells exposed to supernatants from EMP3_KO or EMP3_Scra GL261 cells was measured by ELISA. KO: Knockout; Scra: Scramble. The mean ± S.D. is shown. Ns: nonsignificant, *p < 0.05, **p < 0.01, and ***p < 0.001.


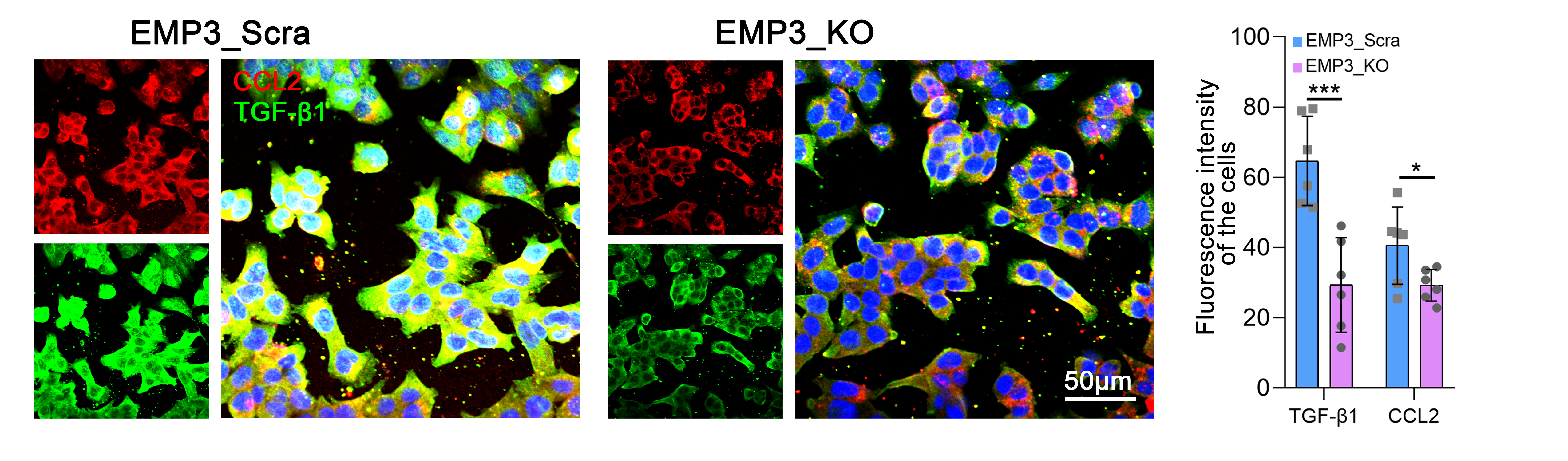


**Supplementary Figure 3. Knockout of EMP3 repressed TGF-β1 and CCL2 secretion in GBM cells.**

Representative images of IF staining for TGF-β1 and CCL2 in EMP3_KO or EMP3_Scra GL261 cells. Scale bar = 50 μm. The histogram summarizes the protein levels of TGF-β1 and CCL2 in GL261 cells by quantification of the fluorescence intensity of the cells. The mean ± S.D. is shown. Ns: nonsignificant, *p < 0.05, **p < 0.01, and ***p < 0.001.


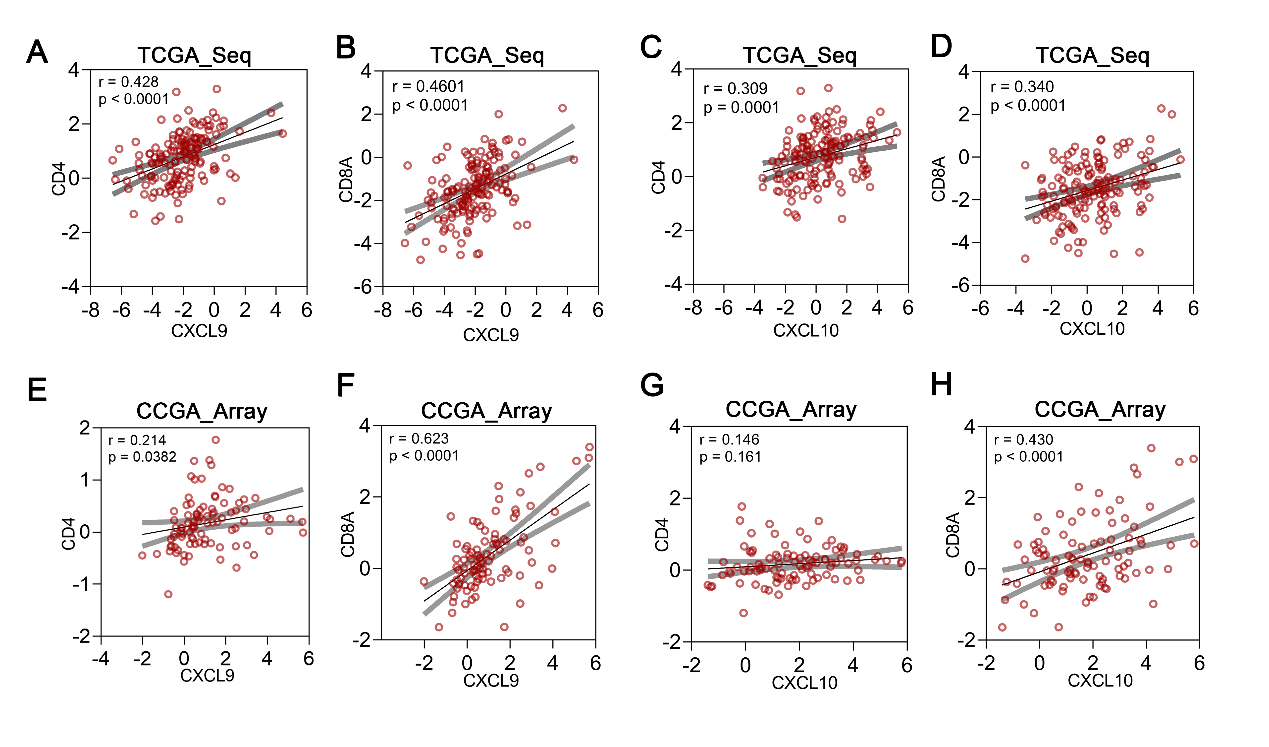


**Supplementary Figure 4. *CXCL9* and *CXCL10* were positively correlated with T cell markers in the datasets.**

A. Pearson correlation plots of the expression of *CXCL9* and *CD4* in the TCGA_seq GBM dataset (n = 153).

B. Pearson correlation plots of the expression of *CXCL9* and *CD8A* in the TCGA_seq GBM dataset (n = 153).

C. Pearson correlation plots of the expression of *CXCL10* and *CD4* in the TCGA_seq GBM dataset (n = 153).

D. Pearson correlation plots of the expression of *CXCL10* and *CD8A* in the TCGA_seq GBM dataset (n = 153).

E. Pearson correlation plots of the expression of *CXCL9* and *CD4* in the CGGA_array dataset (n = 94).

F. Pearson correlation plots of the expression of *CXCL9* and *CD8A* in the CGGA_array dataset (n = 94).

G. Pearson correlation plots of the expression of *CXCL10* and *CD4* in the CGGA_array dataset (n = 94).

H. Pearson correlation plots of the expression of *CXCL10* and *CD8A* in the CGGA_array dataset (n = 94).

**
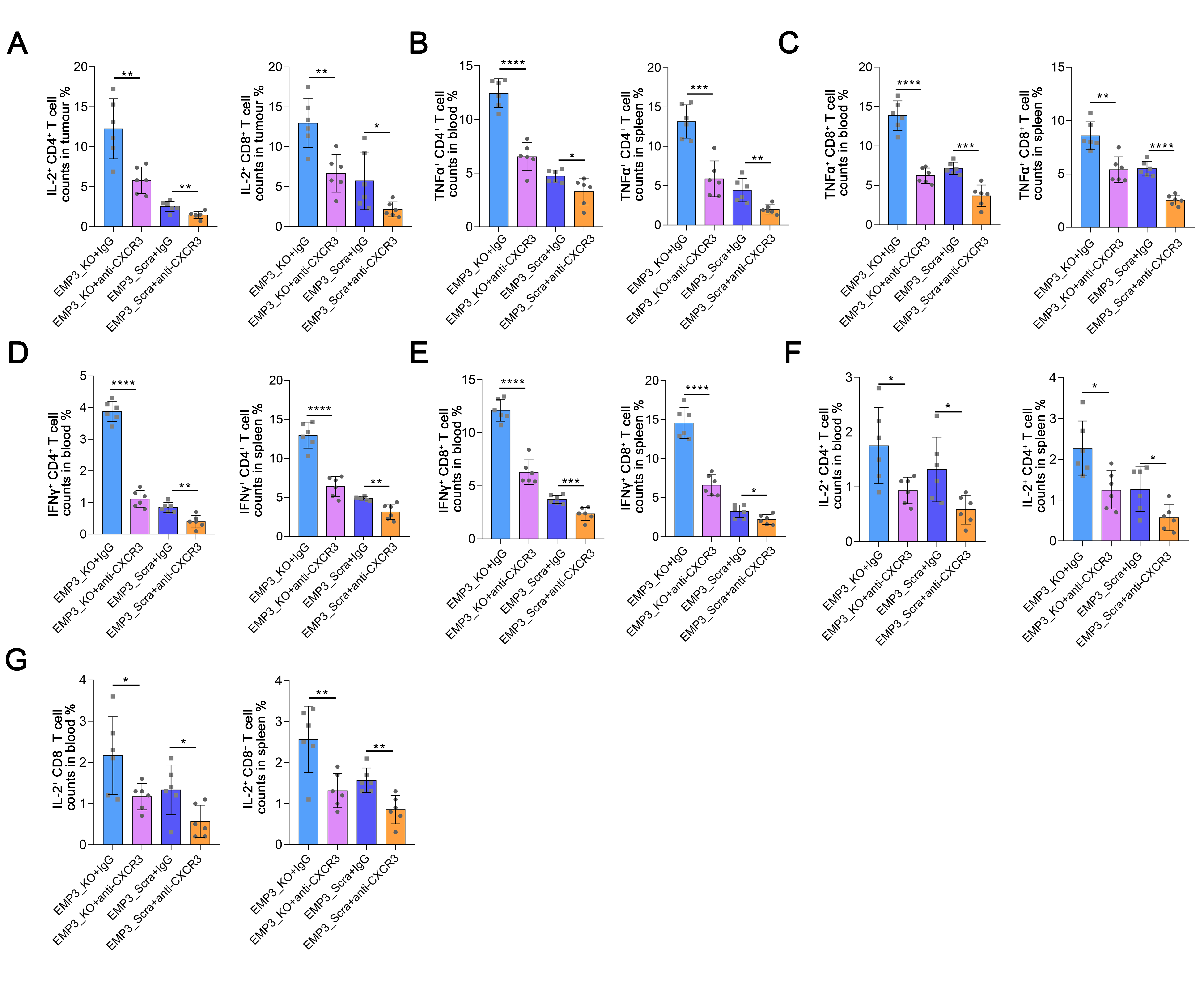
**

**Supplementary Figure 5. The cell count changes of T cells in EMP3_KO tumours treated with an anti-CXCR3 antibody.**

A. Percentages of IL-2^+^ CD4^+^ and IL-2^+^ CD8^+^ T cells in tumours from the EMP3_KO and EMP3_Scra GL261 groups treated with an anti-CXCR3 antibody or isotype IgG. Student’s t-test was performed. KO: Knockout; Scra: Scramble.

B-C. Percentages of TNFα^+^ CD4^+^ and TNFα^+^ CD8^+^ T cells in blood and spleens from the EMP3_KO and EMP3_Scra GL261 groups treated with the anti-CXCR3 antibody or isotype IgG. Student’s t-test was performed. KO: Knockout; Scra: Scramble.

D-E. Percentages of IFNγ^+^ CD4^+^ and IFNγ^+^ CD8^+^ T cells in blood and spleens from the EMP3_KO and EMP3_Scra GL261 groups treated with the anti-CXCR3 antibody or isotype IgG. Student’s t-test was performed. KO: Knockout; Scra: Scramble.

F-G. Percentages of IL-2^+^ CD4^+^ and IL-2^+^ CD8^+^ T cells in blood and spleens from the EMP3_KO and EMP3_Scra GL261 groups treated with the anti-CXCR3 antibody or isotype IgG. Student’s t-test was performed. KO: Knockout; Scra: Scramble. The mean ± S.D. is shown. Ns: nonsignificant, *p < 0.05, **p < 0.01, and ***p < 0.001.


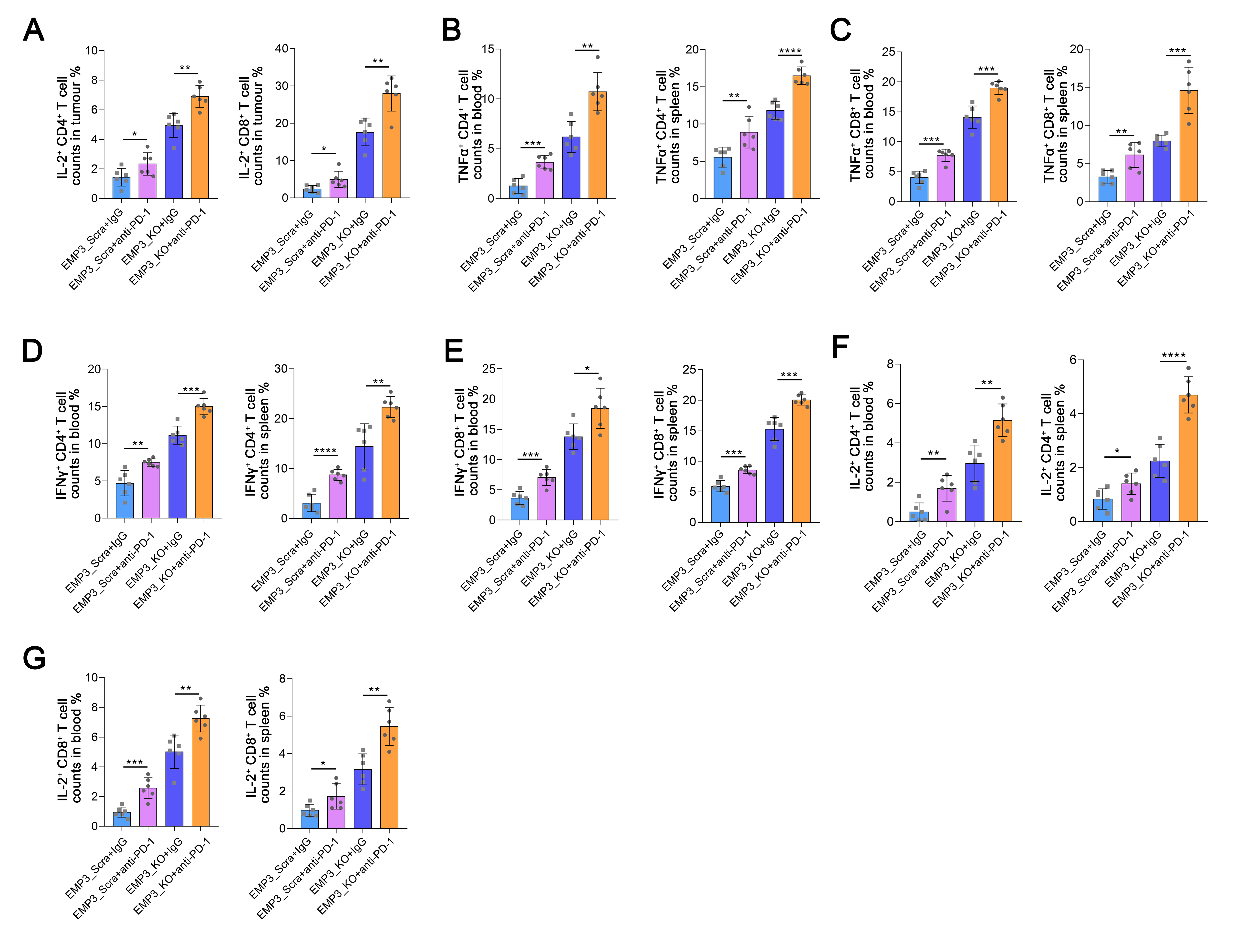


**Supplementary Figure 6. EMP3 inhibition and immune checkpoint blockade mediate T cell activation.**

A. Percentages of IL-2^+^ CD4^+^ and IL-2^+^ CD8^+^ T cells in tumours from the EMP3_KO and EMP3_Scra GL261 groups treated with an anti-PD1 antibody or isotype IgG. Student’s t-test was performed. KO: Knockout; Scra: Scramble.

B-C. Percentages of TNFα^+^ CD4^+^ and TNFα^+^ CD8^+^ T cells in blood and spleens from the EMP3_KO and EMP3_Scra GL261 groups treated with the anti-PD1 antibody or isotype IgG. Student’s t-test was performed. KO: Knockout; Scra: Scramble.

D-E. Percentages of IFNγ^+^ CD4^+^ and IFNγ^+^ CD8^+^ T cells in blood and spleens from the EMP3_KO and EMP3_Scra GL261 groups treated with the anti-PD1 antibody or isotype IgG. Student’s t-test was performed. KO: Knockout; Scra: Scramble.

F-G. Percentages of IL-2^+^ CD4^+^ and IL-2^+^ CD8^+^ T cells in blood and spleens from the EMP3_KO and EMP3_Scra GL261 groups treated with the anti-PD1 antibody or isotype IgG. Student’s t-test was performed. KO: Knockout; Scra: Scramble.

The mean ± S.D. is shown. Ns: nonsignificant, *p < 0.05, **p < 0.01, and ***p < 0.001.

**Supplementary Table 1. Sequences of sgRNAs for Knockout of EMP3 expression.**

| CRISPR Name | Target, PAM & & Flanking Sequence |
| --- | --- |
| Mouse_sgRNA | AAGAGCCCTCCGCGCCGCATGGTGTAGAGTTGGAACATGAAGA |

**Supplementary Table S2. Sequences of the primers used for qRT-PCR**.

| Target | Direction | Sequence |
| --- | --- | --- |
| ARG1 | Forward | 5’-CCACAGTCTGGCAGTTGGAAG-3' |
|  | Reverse | 5'-GGTTGTCAGGGGAGTGTTGATG-3' |
| IL10 | Forward | 5'-AAGGCTATCCTGGTGGAAGAA-3' |
|  | Reverse | 5'-AGGGAAGGGTCAGTCTGTGTT-3' |
| TNF | Forward | 5'-TCTCATGCACCACCATCAAGGACT-3' |
|  | Reverse | 5'-TGACCACTCTCCCTTTGCAGAACT-3' |
| TGFB1 | Forward | 5'-GTACTCCAGAAGACCAGAGG-3' |
|  | Reverse | 5'-AGCTGCTTATCCCAGATTCAGCCA-3' |
| CXCL9 | Forward | 5'-CCGAGGCACGATCCACTACA-3' |
|  | Reverse | 5'-CGAGTCCGGATCTAGGCAGGT-3' |
| CXCL10 | Forward | 5′-GGATGGCTGTCCTAGCTCTG-3′ |
|  | Reverse | 5'-TGAGCTAGGGAGGACAAGGA-3' |
| ACTB | Forward | 5'-GGTCCACACCCGCCACCAG-3' |
|  | Reverse | 5'-CACATGCCGGAGCCGTTGTC-3' |

| **Supplementary Table 3. Clinical data.** | | | |  |
| --- | --- | --- | --- | --- |
| ID | Age | Histology | Gender | IDH_mutation_status |
| 1 | 45 | GBM | Male | Wild-type |
| 2 | 66 | GBM | Female | Wild-type |
| 3 | 54 | GBM | Male | Wild-type |
| 4 | 70 | GBM | Female | Wild-type |
| 5 | 66 | GBM | Female | Wild-type |
| 6 | 36 | GBM | Female | Wild-type |
| 7 | 55 | GBM | Male | Wild-type |
| 8 | 64 | GBM | Male | Wild-type |
| 9 | 32 | GBM | Male | Wild-type |
| 10 | 55 | GBM | Female | Wild-type |
| 11 | 65 | GBM | Male | Wild-type |
| 12 | 67 | GBM | Male | Wild-type |
| 13 | 68 | GBM | Female | Wild-type |
| 14 | 44 | GBM | Female | Wild-type |
| 15 | 69 | GBM | Female | Wild-type |
| 16 | 49 | GBM | Female | Wild-type |
| 17 | 58 | GBM | Female | Wild-type |
| 18 | 52 | GBM | Male | Wild-type |
| 19 | 67 | GBM | Male | Wild-type |
| 20 | 70 | GBM | Female | Wild-type |
| 21 | 47 | GBM | Male | Wild-type |
| 22 | 39 | GBM | Male | Wild-type |
| 23 | 68 | GBM | Male | Wild-type |
| 24 | 63 | GBM | Male | Wild-type |
| 25 | 60 | GBM | Female | Wild-type |
| 26 | 57 | GBM | Female | Wild-type |
| 27 | 54 | GBM | Female | Wild-type |
